# Targeted Temperature Management After Cardiac Arrest: A Systematic Review

**DOI:** 10.7759/cureus.29016

**Published:** 2022-09-11

**Authors:** Aakash Bisht, Ankit Gopinath, Ameer Haider Cheema, Keyur Chaludiya, Maham Khalid, Marcellina Nwosu, Walter Y Agyeman, Ana P Arcia Franchini

**Affiliations:** 1 Internal Medicine, Government Medical College, Amritsar, IND; 2 Internal Medicine, California Institute of Behavioral Neurosciences & Psychology, Fairfield, USA; 3 Internal Medicine, Kasturba Medical College, Manipal, IND; 4 Internal Medicine, Piedmont Athens Regional Medical Center, Athens, USA; 5 Research, California Institute of Behavioral Neurosciences & Psychology, Fairfield, USA

**Keywords:** heart arrest, hypothermia, induced hypothermia, therapeutic normothermia, targeted hypothermia, targeted normothermia, therapeutic hypothermia, targeted temperature management, cardiac arrest, out of hospital cardiac arrest

## Abstract

Targeted temperature management (TTM) has been the cornerstone of post-cardiac arrest care, but even after therapy, neurological outcomes remain poor. We performed a systematic review to evaluate the influence of TTM in post-cardiac arrest treatment, its effect on the neurological outcome, survival, and the adverse events associated with it. We also aimed to examine any difference between the effect of therapy at various intensities and durations on the prognosis of the patient.

A search of two databases was done to find relevant studies, followed by a thorough screening in which the inclusion and exclusion criteria were applied, and a quality appraisal of clinical trials was done. In this systematic review, six randomized clinical trials with a total of 3870 participants were examined. Of these, 2,767 participants were treated with targeted hypothermia to varying degrees (between 31 and 36 degrees Celsius), 931 participants were treated with targeted normothermia (36.5 to 37.5 degrees Celsius), and 172 participants were treated with only normothermia (without any active cooling or interventions).

It was concluded that TTM at a lower temperature did not have any benefit regarding the neurological outcome and mortality over targeted normothermia but was superior to no temperature management. TTM was also found to have significantly more negative effects when the intensity or duration was increased.

## Introduction and background

Cardiac arrest, or heart arrest, is the sudden, sustained loss of mechanical activity of the heart with signs of absence of circulation [1]. It can occur due to underlying cardiac, metabolic, or mechanical causes [2]. Sudden cardiac death is a major worldwide public health concern, accounting for up to 20% of mortality in Western societies [3]. Management of cardiac arrest includes early cardiopulmonary resuscitation and defibrillation, circulatory support, and temperature therapy [4]. Treatment focusing on healing at the molecular level is also gaining traction [5].

Targeted temperature management (TTM) refers to the therapeutic lowering of core body temperature below 36°C, which helps to improve survivability and neurologic outcomes in not just cardiac arrest patients but also in infants with hypoxic-ischemic encephalopathy [6,7]. TTM has been a standard of care for patients with heart arrest since the turn of the century. It was introduced as a treatment guideline by the American Heart Association (AHA) in 2005 after two clinical trials were published in 2002 supporting it [7-9].

Although TTM has been shown to improve prognosis following cardiac arrest, a recently published trial has been shown to differ from this statement [7,10]. Trials published in the previous decade have also failed to show any statistical difference between therapeutic hypothermia at various temperature set points or durations [11,12], leading to more questions being unanswered.

The purpose of this study was to perform a systematic review to assess the current literature and explore if TTM, as the therapy of choice in post-cardiac arrest patients, affects neurologic or functional outcomes and mortality in patients, examine any difference between various intensities and durations of therapy on the prognosis of the patient, and study any adverse events associated with the therapy.

## Review

Methods

A systematic review was conducted based on the Preferred Reporting Items for Systematic Reviews and Meta-Analyses (PRISMA) guidelines using a PRISMA checklist [13]. The search terms included were: cardiac arrest; induced hypothermia; out-of-hospital cardiac arrest; targeted temperature management; therapeutic hypothermia. PubMed’s Medical Subject Headings Tool (MeSH) was used in identifying literature on PubMed. A thorough literature search was conducted on PubMed and Google Scholar. A detailed search strategy has been described in Table 1.

**Table 1:** Keyword search strategy majr - Search tag to find a MeSH heading that is a major topic of an article

Keywords	Database	Results
*Heart arrest *(majr)	PubMed	39,597
*Out-of-hospital cardiac arrest (*majr)	PubMed	5,916
*Hypothermia, induced *(majr)	PubMed	15,833
*Hypothermia, induced/therapeutic use (majr}* or *Hypothermia, induced/therapy (majr)* and *Out-of-hospital cardiac arrest (majr)* or *Heart arrest (majr)*	PubMed	252
*Cardiac arrest* and *Randomized control trial* and *Therapeutic hypothermia* (with a filter of the year of publication since 2000)	Google Scholar	323

The inclusion criteria consisted of English-language literature published from January 1, 2000, till July 16, 2022. The inclusion criteria included literature with free, full-text available, randomized clinical trials with more than 50 subjects over 17 years of age, treatment with mild to moderate hypothermia (30°C to 36°C), patients having an out-of-hospital cardiac arrest (OHCA), any rhythm, and studies reporting pre-determined outcomes of mortality, neurologic outcomes, and adverse effects. Gray literature, no free full-text availability, clinical trials with fewer than 50 patients, treatment with deep hypothermia (30°C), and patients experiencing an in-hospital cardiac arrest (IHCA) were all in the exclusion criteria.

Results

**Figure 1:**
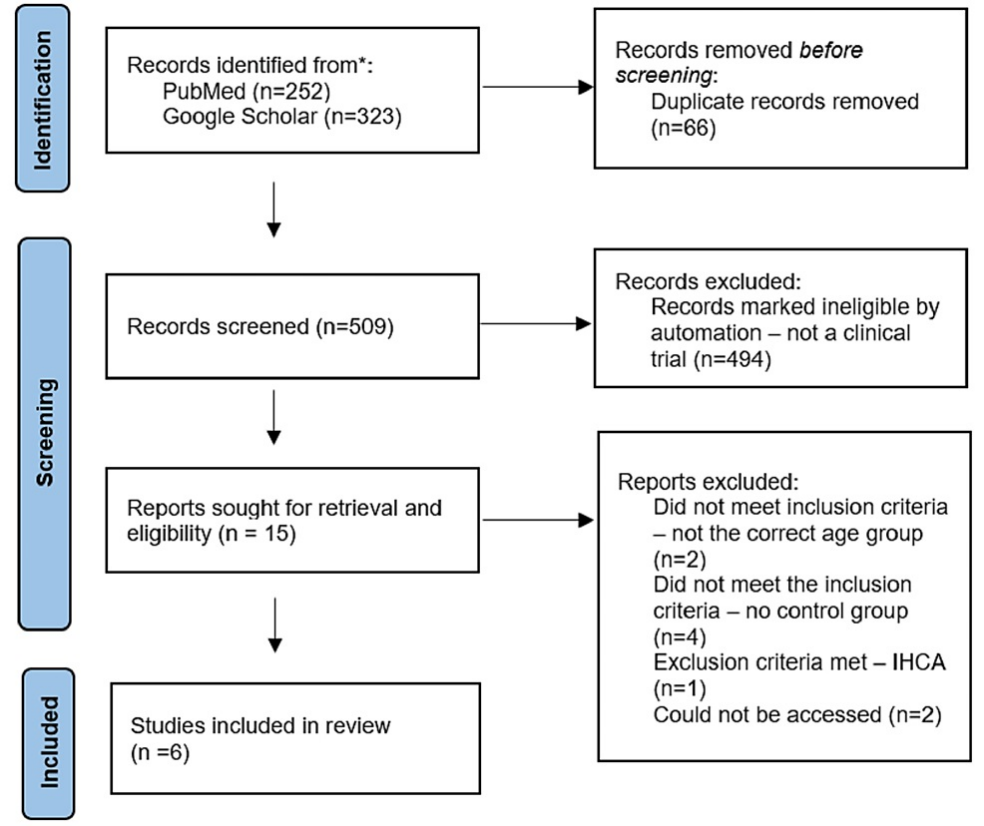
PRISMA flow diagram of the search process. IHCA: In-hospital cardiac arrest

The search process has been detailed in Figure 1. It yielded 252 articles from PubMed and 323 articles from Google Scholar. After 66 duplicate records were removed, the Rayyan automation tool was applied to identify randomized clinical trials, which resulted in 494 records being deemed ineligible [14]. The 15 records were further analyzed independently based on the title, abstract, or full-text and were assessed for eligibility. Seven studies either met one or more of the exclusion criteria or did not meet the inclusion criteria set, and two studies could not be accessed. Quality assessment was done with the Risk of Bias 2.0 (RoB) tool developed by Cochrane, and the four studies reported a low risk of bias and two studies reported some concerns. [15]. All six randomized clinical trials were included in this systematic review. A detailed summary has been included in Table 2.

**Table 2:** Results of the Cochrane Risk of Bias assessment tool TTM2: Targeted Temperature Management-2, TTH48: Time-differentiated Therapeutic Hypothermia, TTM: Targeted Temperature Management, HACA: Hypothermia After Cardiac Arrest

Trial	Domain 1	Domain 2	Domain 3	Domain 4	Domain 5	Risk of Bias
TTM2 trial (2021) [10]	Low	Low	Low	Low	Low	Low
CAPITAL CHILL trial (2021) [16]	Low	Low	Low	Low	Low	Low
TTH48 trial (2017) [12]	Low	Low	Low	Low	Low	Low
TTM trial (2013) [11]	Low	Low	Low	Low	Low	Low
HACA trial (2002) [9]	Low	Some concern	Low	Low	Low	Some concern
Trial by Bernard et al. (2002) [7]	Low	Some concern	Low	Low	Low	Some concern

The six randomized clinical trials had a total of 3870 subjects, of which 2,767 participants were treated with targeted hypothermia to varying degrees (between 31 and 36 degrees Celsius), 931 participants were treated with targeted normothermia (36.5 to 37.5 degrees Celsius), and 172 participants were treated with only normothermia (without any active cooling or interventions). Out of the 2767 subjects treated with targeted hypothermia, 184 were treated at 31°C, 1934 were treated at 33°C, 183 were treated at 34°C, and 466 were treated at 36°C. All the trials reported the technique of cooling the patients, which included surface and invasive cooling devices. All trials had a follow-up period of six months or more except one that had a follow-up period until discharge [7].

Discussion

TTM has been the cornerstone of post-cardiac arrest care since 2005. Most recently, the 2020 AHA guidelines for post-cardiac arrest care and the 2022 European Society of Intensive Care Medicine (ESICM) and European Resuscitation Council (ERC) guidelines for critical care have included procedures and recommendations for physicians to follow [17,18]. The 2020 AHA guidelines stated that TTM between 32°C and 36°C for at least 24 hours is advised. The 2022 ERC-ESICM guidelines recommended continuous monitoring of core body temperature and preventing pyrexia for at least 72 hours in patients who remain comatose after a cardiac arrest. The guidelines also recommended temperature control with antipyretic medications or cooling devices and warned against using pre-hospital cooling with the infusion of cold fluids.

The Targeted Temperature Management-2 (TTM2) trial was the largest trial conducted to date, across 14 countries with more than 1800 subjects, and published in 2021 [10]. It compared the effect of targeted hypothermia at 33°C to targeted normothermia at 37.5°C. The temperatures were maintained with a surface or intravascular temperature management device for 28 hours after randomization, followed by rewarming to 37°C at 0.3°C per hour for 72 hours after randomization. The trial concluded that therapeutic hypothermia did not lead to lower mortality than therapeutic normothermia, and a definitive improvement in neurological outcome could not be achieved as measured by the modified Rankin scale. There were several limitations to the trial, which included a treatment protocol of sedation, paralysis, and mechanical ventilation in all patients, irrespective of their prognostic status. It could be inferred that several adverse effects such as pneumonia or sepsis could be a result of these interventions in the control group. The risk of arrhythmia resulting in hemodynamic compromise was noted to be higher in patients treated with the hypothermia protocol rather than the normothermia protocol (relative risk = 1.45, 95% CI = 1.21-1.75, p<0.001). This was in line with the known finding that moderate hypothermia is pro-arrhythmic [19].

The CAPITAL CHILL trial was published in 2021 with 389 subjects to evaluate if targeted hypothermia at lower temperatures would yield better results [16]. Hypothermia at 31°C and 34°C was maintained with active cooling with an endovascular cooling catheter for 24 hours, followed by active rewarming at 0.25°C/h till 37°C was reached. The study was conducted for 87 months in a tertiary cardiac care center in Canada and concluded that no difference was found between the two groups in terms of neurologic outcomes, mortality, and serious adverse events. This trial followed a protocol of pre-hospital cooling, which was done with ice packs only. The risk of bias was greatly reduced as it was the first double-blind trial to be done in its domain, but it was reported that the study may have been underpowered to detect any clinically critical difference as the study was based on a 15% absolute risk reduction.

The Time-differentiated Therapeutic Hypothermia (TTH48) trial, which included 355 participants, was the first of its kind, comparing the effects of TTM at 33°C for 48 hours to TTM at 24 hours with surface or invasive cooling methods followed by rewarming at 0.5°C per hour until 37°C was reached [12]. It was conducted across 6 countries and concluded that treatment for 48 hours did not significantly improve the 6-month neurological outcome. However, the study did report a significantly increased risk of any adverse event occurring in the 48-hour group compared to the 24-hour group (97% vs. 91%, relative risk = 1.06, 95% CI = 1.01-1.12, p = 0.03), which supports the data that the use of therapeutic hypothermia has many adverse effects [20]. Furthermore, the risk of hypotension was reported to be significantly higher in the 48-hour group (p = 0.013), while the risk of bleeding was significantly higher in the 24-hour group (p = 0.03).

The TTM trial was published in 2013 with 950 subjects across 10 countries and compared the outcomes of TTM at 33°C and 36°C [11]. Target temperatures were maintained with surface or intravascular cooling devices for 28 hours, followed by rewarming to 37°C at 0.5°C per hour, followed by maintenance of temperatures below 37.5°C for 72 hours. It was the first trial in which active rewarming at a pre-specified rate was done following therapeutic hypothermia. This trial concluded there were no significant benefits in outcomes at the two target temperatures, although it did report lower mortality than the Hypothermia After Cardiac Arrest (HACA) trial. The groups in the two studies were not comparable. Hypokalemia was the only adverse effect that occurred significantly more often in the 33°C group than in the 36°C group (19% vs. 13%, P = 0.02). Other adverse events reported were seizures, including myoclonic and tonic-clonic seizures, major bleeding, pneumonia, severe sepsis, septic shock, various types of arrhythmias, electrolyte disorders, and renal replacement therapy.

The HACA trial was a multicenter randomized controlled trial conducted across 5 European countries with 275 subjects [9]. It was considered the landmark trial following which TTM was introduced as a standard of care in post-cardiac arrest care. Hypothermia was maintained between 32°C and 34°C with an external cooling device for 24 hours followed by passive rewarming over eight hours, while normothermia was maintained in a conventional hospital setting without any active cooling intervention. The trial concluded that patients treated with TTM had a statistically better neurological outcome (95% CI 1.08-1.81, p = 0.009) and reduced mortality rate (95% CI 0.58-0.95, p = 0.02) than patients having standard intensive care unit (ICU) care without any temperature management. The trial revealed a trend of higher infectious complications (pneumonia and sepsis) in the hypothermia group. This trial had a substantial risk of bias [21,22]. The trial was performed on patients with cardiac arrest having an initial rhythm of ventricular fibrillation only, and thus the results could not be generalized to all cardiac arrest patients. Also, significant hyperthermia was seen in the normothermic group, which is detrimental to survival [23]. The study did not report a statistical difference in adverse events between the two groups.

Bernard et al. [7] published a trial in 2002 that consisted of 77 subjects in Australia. Hypothermia was initiated by basic cooling measures in the ambulance, followed by active surface cooling in the hospital till the core body temperature reached 33°C. The target temperature was maintained for 12 hours, followed by active external rewarming for six hours until 24 hours after the presentation. Normothermia was maintained at a core target temperature of 37°C for 24 hours without any active cooling intervention. The study was conducted for 34 months, and only patients that presented with an initial cardiac rhythm of ventricular fibrillation were included in the trial. The trial concluded that TTM significantly improved outcomes following cardiac arrest (95% CI = 0.13-0.43, p = 0.046). The study may have a high risk of bias as patients with poor prognoses were excluded from the trial. Also, the follow-up period was limited to discharge from the hospital only and did not include post-hospital follow-up. The studies are summarized in Table 3.

**Table 3:** Summary of randomized control trials TTM2: Targeted Temperature Management-2, OHCA: Out-of-hospital cardiac arrest, CI: Confidence Interval, p: probability value, TTH48: Time-differentiated Therapeutic Hypothermia, TTM: Targeted Temperature Management, GCS: Glasgow Coma Scale, HACA: Hypothermia After Cardiac Arrest

Trial	Population	Neurologic or functional outcome	Mortality outcome	Significant adverse events
TTM2 trial (2021) [10]	1861 subjects were included. The patients had an OHCA and were unconscious at admission. Patients with unwitnessed asystole were excluded.	55% of patients in the hypothermia group and 55% of patients in the normothermia group had moderately severe disability or worse at 180 days on the modified Rankin scale.	50% of patients in the hypothermia group and 48% of patients in the normothermia group had died by day 180.	Arrhythmia resulting in hemodynamic compromise was found in 24% of hypothermia patients and 16% of normothermia patients (95% CI= 1.21-1.75 p<0.001). Other adverse events reported were pneumonia, sepsis, bleeding, and skin complications.
CAPITAL CHILL trial (2021) [16]	367 subjects were included. Patients had an OHCA and were comatose at admission. Patients with unwitnessed asystole were excluded.	48.4% of patients in the moderate hypothermia group and 45.4% of patients in the mild hypothermia group had poor neurologic outcomes or worse at 180 days on the modified Rankin Scale.	43.5% of patients in the moderate hypothermia group and 41% of patients in the mild hypothermia group had died by day 180.	Non-significant adverse events reported were stroke, seizures, renal replacement therapy, pneumonia, cardiogenic shock, stent thrombosis, deep vein, inferior vena cava thrombi, recurrent cardiac arrest, and arrhythmia.
TTH48 trial (2017) [12]	351 subjects were included. Participants had an OHCA with shockable and non-shockable rhythms. Patients with unwitnessed asystole were excluded.	31% of patients in hypothermia for the 48-hour group and 35% of patients in hypothermia for the 24-hour group had poor neurologic outcomes or worse on the cerebral performance category scale at six months.	27% of patients in hypothermia from the 48-hour group and 34% of patients in hypothermia from the 24-hour group had died by 6 months.	The risk of any adverse event was 97% in the 48-hour group compared to 91% in the 24-hour group (95% CI= 1.01-1.12 p=0.03). Hypotension was found in 62% of patients in the 48-hour group and 49% of patients in the 24-hour group (P=0.013). Severe bleeding was found in 1% of patients in the 48-hour group and 4% of patients in the 24-hour group (p=0.03). Other adverse events reported were new pupil abnormalities, seizures, arrhythmias, renal replacement therapy, pneumonia, and sepsis.
TTM trial (2013) [11]	939 subjects were included. Patients had an OHCA, irrespective of rhythm, and were unconscious with GCS<8. Patients with unwitnessed asystole were excluded.	54% of patients in the 33^o^C group and 52% of patients in the 36^o^C group had poor neurologic outcomes or worse on the cerebral performance category scale at day 180. 52% of patients in both, the 33^o^C group and 36^o^C group had poor neurologic outcomes or worse on the modified Rankin scale at day 180.	50% of patients in the 33^o^C group and 48% of patients in the 36^o^C group had died by the end of the trial (mean follow-up was 256 days).	Hypokalemia was found in 19% of patients in the 33^o^C group and 13% of patients in the 36^o^C group. (p=0.02) Other adverse events reported were seizures, bleeding, infections, arrhythmias, renal replacement therapy, and electrolyte disorders.
HACA trial (2002) [9]	275 subjects were included. Patients had witnessed OHCA with a shockable rhythm.	At six months, 55% of hypothermia patients and 39% of normothermia patients had a favorable neurologic outcome based on the Pittsburgh cerebral performance category; this was statistically significant (95% CI = 1.08-1.81, p = 0.009).	41% of patients in the hypothermia group and 55% of patients in the normothermia group had died by six months; this was statistically significant (95% CI = 0.58-0.95, p = 0.02).	Non-significant adverse events reported were bleeding, pneumonia, sepsis, pancreatitis, renal failure, pulmonary edema, seizures, arrhythmias, and pressure sores.
Trial by Bernard et al. (2002) [7]	77 subjects were included. Patients had OHCA with an initial rhythm of ventricular fibrillation and persistent coma after the return of spontaneous circulation.	49% of patients in the hypothermia group and 26% of patients in the normothermia group had good neurologic outcomes at discharge; this was statistically significant (95% CI = 0.13-0.43, p=0.046).	51% of patients in the hypothermia group and 68% of patients in the normothermia group had died by discharge.	No clinically significant adverse events were reported.

Outcomes

As described above, therapeutic hypothermia at different temperatures or durations did not confer any neurological or mortality benefit for post-cardiac arrest patients in four out of the six trials. The remaining two trials had a substantial risk of bias and thus have been regarded as having a low level of evidence. However, if the risk of bias was removed, a hypothesis could be made that targeted temperature management at any set point (31°C - 36.5°C) is better than normothermia progressing to hyperthermia (>37.5°C). The risk of any adverse event occurring increased with the intensity and duration of hypothermia in three trials. The most common adverse events due to therapy reported, but not limited to, were: seizures, pneumonia, various arrhythmias, severe bleeding, renal replacement therapy, sepsis of any kind, thrombosis, hypotension, and recurring cardiac arrest.

Other Studies

Trials that have contributed immensely to our understanding of TTM in post-arrest care include the Therapeutic Hypothermia After Cardiac Arrest in Nonshockable Rhythm (HYPERION) trial, the Finding the Optimal Cooling Temperature After Out-of-Hospital Cardiac Arrest (FROST-1) trial, the Therapeutic Hypothermia After Pediatric Cardiac Arrest Out-of-Hospital (THAPCA-OH) trial, and the Therapeutic Hypothermia After Pediatric Cardiac Arrest In-Hospital (THAPCA-IH) trial [24-27]. The HYPERION trial was published in 2019 but was not included in the systematic review as it included IHCA patients [24]. Although the trial concluded that TTM has a better neurological outcome than normothermia in patients presenting with a non-shockable rhythm, it had a fragility index of only 1.0, which meant that if there was a single change in the outcome of the hypothermia group, the results would be nonsignificant. Also, in the subgroup analysis of the study, it was found that the majority of the patients that had a better outcome were from the IHCA group rather than the OHCA group, and a significant number of patients developed a fever in the normothermia group. Real-world statistics show that cardiac arrests occur more commonly in out-of-hospital settings than in hospitals, and thus these considerations should be kept in mind when determining TTM as a therapeutic guideline for OHCA patients [28]. On the other hand, FROST-1 was a multi-center trial published in 2018 but was not included in the systematic review as the full text could not be accessed [25]. The trial was done to compare the effects of therapeutic hypothermia at 32°C, 33°C, and 34°C. Based on the abstract, it was concluded that no significant difference in the neurological outcome could be achieved, as was found in the CAPITAL CHILL trial of 2021. The THAPCA-IH and THAPCA-OH trials were not included in this systematic review as these trials included the pediatric population [26,27]. The THAPCA-IH trial was published in 2017 and had 329 subjects but was stopped prematurely due to the futility of the study. The trial concluded that no statistical significance could be achieved in determining whether therapeutic hypothermia was superior to therapeutic normothermia in terms of mortality and favorable neurological outcome. The THAPCA-OH trial was published in 2015 and had 295 subjects. This trial also had a similar conclusion to the THAPCA-IH trial.

Analogous studies conducted in animals were shown to prove that TTM had a favorable effect on normothermia and had a dose-dependent relationship reported as a lower temperature and better neurological outcomes [29,30]. Such similar effects have not been reproduced in human physiology as the effect of TTM depends on many factors [31]. Various studies have shown that markers such as pro-calcitonin, serum lactate, S-100B, and neuron-specific enolase in combination with other biomarkers can be used to predict the outcome following a cardiac arrest. [32-36]

Limitations

The search strategy for this systematic review was limited to two databases. Only free, full-text, randomized trials were included, possibly leading to other studies being omitted. Another limitation was that only English language studies were included. The generalizability was restricted to OHCA only as IHCA patients would have better access to medical interventions, which would affect the outcome as a whole.

## Conclusions

TTM has been an established treatment protocol for almost two decades. However, its benefits have not yet been proven beyond doubt. Studies in the past decade have not been able to identify any advantage of targeted hypothermia over targeted normothermia. Even in the HACA trial, hypothermia was reported to be statistically significant over normothermia as fever was not controlled.

In light of the current literature, we conclude that targeted hypothermia at different intensities and durations yields no better results than targeted normothermia but may cause significant adverse effects in patients. In consideration of the above findings, a blanket optimal target temperature cannot be used for all patients with cardiac arrest as several clinical variables play a part in the success of TTM currently. Physicians may find it favorable to treat patients at targeted normothermia (36°C to 37°C). Future studies should be conducted to study whether interventions to prevent pyrexia only (TTM vs. targeted normothermia vs. no temperature control) may improve long-term survival, preferably with an assessment of markers that have shown promising results. To assess true outcomes, studies should be conducted with a longer follow-up period. 
